# Subicular circuit in epilepsy: deconstruct heterogeneity for precise therapeutics

**DOI:** 10.3389/fnins.2023.1202372

**Published:** 2023-06-13

**Authors:** Ying Wang, Mengru Liu, Qingyu Wang

**Affiliations:** ^1^Institute of Neuropsychiatric Diseases, The Affiliated Hospital of Qingdao University, Qingdao University, Qingdao, China; ^2^School of Basic Medicine, Qingdao University, Qingdao, China; ^3^Department of Anesthesiology, The Affiliated Hospital of Qingdao University, Qingdao University, Qingdao, China

**Keywords:** epilepsy, subiculum, circuit, heterogeneity, HCN channel

## 1. Introduction

Temporal lobe epilepsy (TLE) is the most common form of epilepsy, accounting for more than half of adult patients (Kanner and Bicchi, 2022). The dysfunction of the hippocampus-centric network has been generally recognized as a critical pathogenic factor of TLE (Wang and Chen, 2019). The hippocampus itself is composed of several sub-regions, including the cornu ammonis (CA1-4), the dentate gyrus, and the subiculum (Knierim, 2015). Among them, the subiculum (SUB), a gating of hippocampal output circuits (O'Mara et al., 2001), is widely perceived as a site of ictogenesis and has been attached serious attention in TLE (Fei et al., 2021). The subiculum mainly contains pyramidal projection neurons and GABAergic interneurons (O'Mara et al., 2001). The diverse roles of different subicular GABAergic subtypes in epilepsy and the underlying microcircuit mechanisms have been reported in previous studies (Wang et al., 2017). Chen's lab and other groups have devoted themselves to dissecting the role of the subiculum in epilepsy. They identified the subiculum as a potential target for deep brain stimulation in the clinical treatment of TLE and revealed the role of local GABAergic and pyramidal neurons in the expression of the secondary generalized seizure (sGS) and drug resistance in the past several years (Cohen et al., 2002; Zhong et al., 2012; Wang et al., 2017; Xu et al., 2019). However, the underlying neural circuit mechanisms are still rarely known. Recently, they have made new progress focusing on the long-projecting discrete subicular output circuits and demonstrated their diverse roles in the generation of hippocampal seizures, which further confirmed the pivotal position of the subiculum in TLE (Fei et al., 2022).

## 2. Manuscript formatting

### 2.1. HCN channel in ANT-projecting subicular pyramidal neurons contributed to hippocampal seizures

Fei et al. (2022) proved that subicular pyramidal neurons were activated in hippocampal seizures, especially in sGS, whereas it is exciting that sGS-induced c-Fos activation showed regional specificity in the deep layer of the subiculum. The heterogeneity of subicular pyramidal neurons has been indicated in previous studies (Cembrowski et al., 2018), where subiculum pyramidal cells are divisible into proximal and distal subclasses and functionally differential in spatial working memory. In this study, the authors paid more attention to the deep and superficial dissociation and clearly verified their structural and functional heterogeneity in sGS as never before. Then, they focused on whether the role of heterogeneous subicular pyramidal neurons in epilepsy is mediated by diverse circuits. The authors paid attention to the anterior nucleus of the thalamus (ANT). Retrograde tracing showed that ANT-projecting neurons are almost completely located in the deep layer of the subiculum which is structurally consistent with the regional specificity of sGS-induced c-Fos activation. Moreover, behavioral studies showed that optogenetic activation of ANT-projecting subicular pyramidal terminals could promote, while inhibition could retard, the development and expression of hippocampal seizures in both acute and chronic epileptic models. Therefore, it so far indicated that ANT-projecting subicular pyramidal neurons largely contributed to the generation of hippocampal seizures, especially sGS. ANT has been believed to play a critical role in generalized seizures (Wang et al., 2016), and has been clinically used as an effective target for deep brain stimulation in the treatment of epilepsy (Theodore and Fisher, 2004; Yu et al., 2018). Therefore, the SUB-ANT pyramidal circuit probably plays a crucial role in the generalization and amplification of hippocampal seizures.

To further investigate the inner mechanism underlying the “amplifier” role of the SUB-ANT circuit in hippocampal seizures, the authors focused on their specific electrophysiological properties. ANT-projecting subicular neurons showed a higher proportion of burst firing, and these bursting neurons exhibited a higher sag ratio at hyperpolarizing injected currents and larger *I*_h_ density, which are generally mediated by the somatic hyperpolarization-activated cyclic nucleotide-gated cation (HCN) channel. Moreover, selectively knocking down the HCN1 in ANT-projecting subicular neurons could decrease their bursting activity and alleviate hippocampal seizures. In fact, numerous previous studies have discussed the role of HCN channel and *I*_h_ in epilepsy, but no consensus was reached (Noam et al., 2011), which might be caused by the region- and cell type-specific function of different HCN channel isoforms in epilepsy. Unprecedentedly, Fei et al. (2022) verified that strengthened HCN channel activity in ANT-projecting subicular pyramidal neurons specifically contributed to transmitting epileptic signals and facilitating seizure generalization via enhancing bursting activity and synaptic plasticity, which is of great significance. It is worth noting that ANT-projecting subicular pyramidal neurons might have other parallel projections, which might also impact seizure generation and need further concern. Moreover, the HCN channel also contributes to post-inhibitory action potential firing. Thus, the rebound bursting property underlying ANT-projecting subicular pyramidal neurons might also contribute to seizure amplification. In addition to the HCN channel, we cannot deny other molecular mechanisms underlying the enhanced bursting activity of ANT-projecting subicular neurons, such as sodium and calcium channels. Therefore, it would be better to combine transcriptomic analysis based on specific circuits.

### 2.2. Heterogenous subicular circuits transmit different information during the epileptic condition

In addition, the authors also dissected the role of other subicular pyramidal output pathways. They found that entorhinal cortex (EC)-projecting neurons mostly collected in the superficial layer of the subiculum, and their activity showed a long-term decline during both FS and sGS even after seizure termination. Meanwhile, the inhibition of EC-projecting subicular pyramidal terminals aggravated, but activation had no effect on hippocampal seizures. Therefore, the distribution and function were totally different between SUB-ANT and SUB-EC pyramidal circuits. It is generally acknowledged that the imbalance of excitation and inhibition is the primary cause of epileptogenesis, and the inhibition of glutamatergic transmission or promoting GABAergic transmission has been used to retard seizures (Wang et al., 2018). However, EC is a unique area and does just the opposite in hippocampal seizures. It has been reported that optogenetic inhibition of EC pyramidal neurons resulted in a higher propensity of epileptic behaviors while optogenetic activation retarded hippocampal seizures by inducing the GABA-mediated inhibition of hippocampal neurons (Xu et al., 2016). It can be speculated that the opposite role of the SUB-EC and SUB-ANT pyramidal circuit in epilepsy might be mediated by different downstream circuit mechanisms. However, the downstream mechanism underlying the SUB-EC pyramidal circuit was not further explored here. Apart from that, the authors also rationale such opposite as distinct upstream efferent of ANT- and EC-projecting pyramidal neurons in their discussion, which could actually be testified by RV-based retrograde tracing in further studies. Moreover, modulation of SUB-NAc/MMB pyramidal circuits didn't impact hippocampal seizures. These results indicate that the role of subicular pyramidal neurons in epilepsy is circuit-specific and distinct downstream circuits transmit different information during epileptic conditions.

### 2.3. Perspective

**Figure 1:**
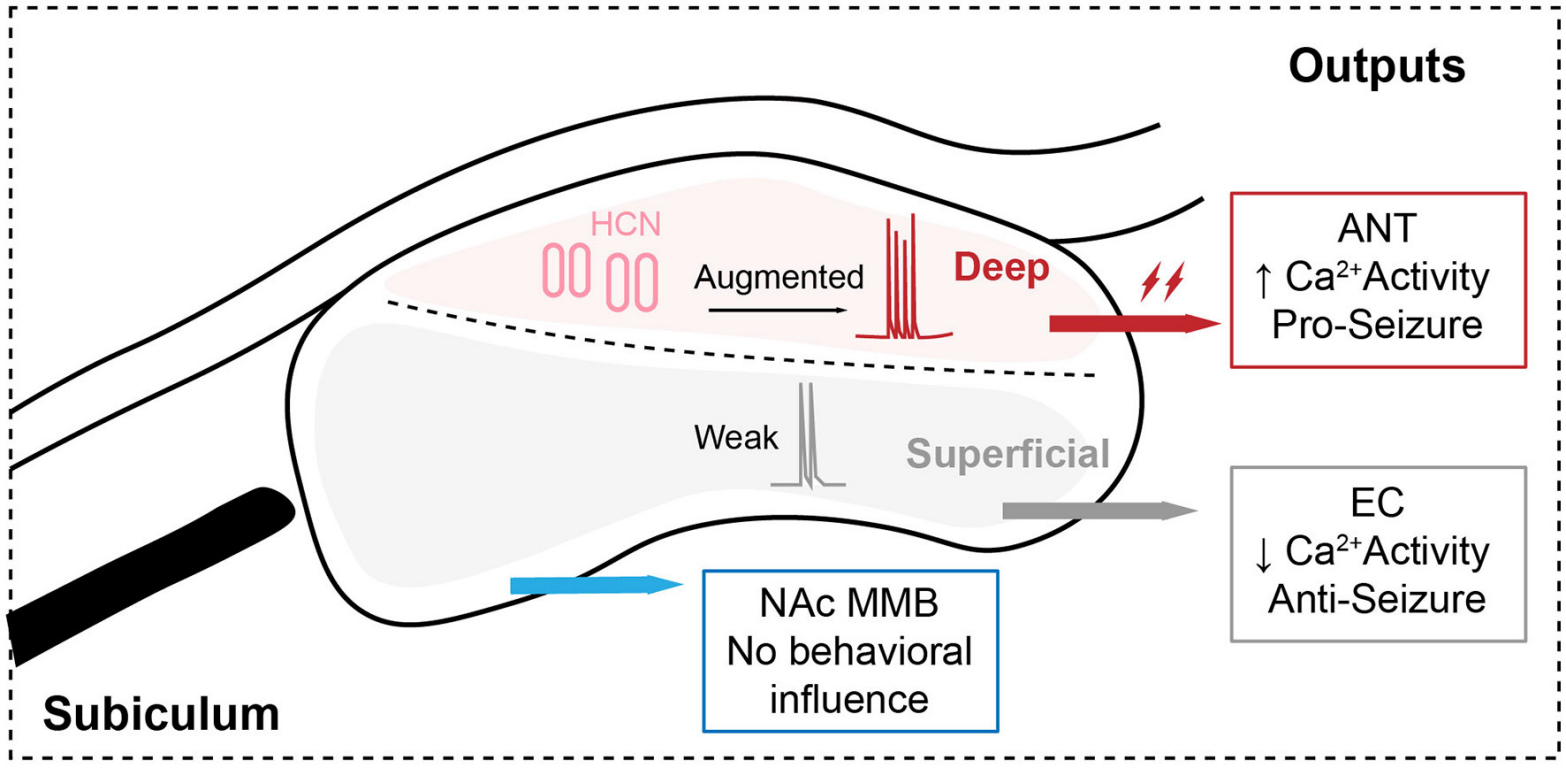
Structrally and functionally heterogenous subicular pyramidal circuits control the generalization of hippocampal seizures.

Overall, Fei et al. (2022) comprehensively and deeply dissected the structurally and functionally heterogeneous subicular pyramidal circuits. Their results showed that ANT-projecting subicular pyramidal neurons are defined as an “amplifier” for controlling the generation of hippocampal seizures *via* enhanced HCN-related bursting (Figure 1), which might be of therapeutic significance in the clinic. Certainly, it also leaves many questions behind (1). Given that the heterogeneous subicular circuits respond to hippocampal seizures in different ways, there might be diverse upstream innervations of the subiculum, which is worthy of further investigation *via* retrograde viral tracing (2). Although the sub-ANT pyramidal projection has been demonstrated to obviously promote hippocampal seizures and act as a crucial circuit for precise seizure control, its functional characteristics are not clear. The authors only used 20 Hz blue light as the parameter for optogenetic activation. However, considering the participation of the HCN channel, “bursting” stimulation of the SUB-ANT pyramidal circuit might be more effective, which can be tried by the rebound activation with eNPHR3.0 (Yang et al., 2018) (3). The authors did not verify the alteration of the HCN channel during the development of hippocampal seizures, especially the propagation from FS to GS, which is important for evaluating the HCN channel as a potential anti-seizure drug (4). The subiculum is closely involved in cognitive function. Therefore, it needs further research to find whether subicular modulation is safe for cognition, which is often destroyed in epilepsy. Together, deconstructing heterogeneity of the subicular circuit in epilepsy is critically important for precise therapeutics. We expect more development and improvement in the subiculum-related work, which would finally be beneficial for translational medicine.

## Author contributions

YW read the relevant literature and wrote the manuscript with support from ML and QW. All authors contributed to the article and approved the submitted version.
